# Bullous pemphigoid and milia: prevalence and clinical laboratory findings in a Brazilian sample^☆^

**DOI:** 10.1016/j.abd.2021.10.003

**Published:** 2022-05-27

**Authors:** Sebastián Vernal, Ederson Valei de Oliveira, Roberto Bueno Filho, Tamiris A. Julio, Eduardo A. Donadi, Aline Turatti, Norito Ishii, Takashi Hashimoto, Ana Maria Roselino

**Affiliations:** a Laboratory of Dermatology, University Hospital, Faculdade de Medicina de Ribeirão Preto, Universidade de São Paulo, Ribeirão Preto, SP, Brazil; b University Hospital, Dermatology Division, Department of Clinical Medicine, Faculdade de Medicina de Ribeirão Preto, Universidade de São Paulo, Ribeirão Preto, SP, Brazil; c Immunology Division, Department of Clinical Medicine, Faculdade de Medicina de Ribeirão Preto, Universidade de São Paulo, Ribeirão Preto, SP, Brazil; d Department of Dermatology, Kurume University School of Medicine and Kurume University Institute of Cutaneous Cell Biology, Fukuoka, Japan

**Keywords:** Hemidesmosomal plaque protein, HLA antigens, Pemphigoid, bullous, 230 kDa protein

## Abstract

**Background:**

Bullous pemphigoid (BP) associated with milia lesions has been increasingly reported, but its prevalence has not been reported in a Brazilian BP population yet.

**Objectives:**

To describe the occurrence and clinical-laboratorial findings of BP-milia association in a southeastern Brazilian sample.

**Methods:**

A descriptive study based on the medical charts of 102 BP patients was accomplished. Clinical and laboratory data of BP-milia patients were compiled. Total serum IgE measurements, immunoblot assays based on basement membrane zone antigens, and HLA-DQ alleles typing were performed.

**Results:**

Milia was evident in 8 (7.8%) BP patients, five males, aged between 46 and 88 years. Increased total IgE levels were determined in 7 (87.5%) of the eight patients. In five of eight patients, immunoblotting showed IgG reactivity against the BP180-NC16a domain but not against collagen VII or laminin-332; it also revealed reactivity against the BP180 C-terminal domain or LAD-1, or both in four of them. The HLA-DQB1*03:01 and HLA-DQA1*05:05 alleles were identified in three of five BP-milia patients. Moreover, three of five cases presented the HLA-DQB1*06 allelic group.

**Study limitations:**

HLA determination was performed in five patients.

**Conclusions:**

Milia formation in BP patients seems to be less uncommon than previously admitted. Laboratory data revealed increased IgE; autoantibodies against the BP180 C-terminal domain or LAD-1, or both; and the HLA-DQB1*06 allelic group, described for the BP-milia association. Careful determination of antibodies against basement membrane zone molecules and HLA characterization in different populations may provide further insights into this association.

## Introduction

Bullous pemphigoid (BP) is the most prevalent autoimmune bullous disease worldwide. It is clinically characterized by pruritus, tense bullae, and urticarial patches on the skin; blistering and erosions in the mucous membranes are less frequent. Subepidermal blisters with predominant eosinophilic inflammatory infiltrate in the upper dermis are evident on histopathological biopsy. Autoantibodies against hemidesmosome proteins BP180 (also termed BPAG2 or type collagen XVII) or BP230 (also termed BPAG1 or dystonin), or both, are shown in serum samples.^1^

BP is known to be associated with HLA alleles and environmental factors.^2^, ^3^, ^4^ Of interest, its clinical presentation has been associated with milia on scar of blistering lesions or in non- lesioned skin in certain BP patients.^5^ Milia are mainly associated with Epidermolysis Bullosa Acquisita (EBA) and porphyria cutanea tarda.^6^, ^7^ Prost et al. (1987) described the presence of milia in cases of EBA and cicatricial pemphigoid, but milia were absent in the analyzed BP patients.^8^ Reports on BP-milia association have been increasing in the last decades in a diverse populations, but its prevalence and clinical-laboratory data have not been reported in a Brazilian BP population yet.^9^, ^10^, ^11^, ^12^, ^13^, ^14^, ^15^

Of 102 compiled BP cases, we report eight patients who presented associated milia. Clinical, laboratory and blotting assays, and HLA-DQ alleles determination were assessed in these eight BP-milia patients.

## Methods

This descriptive study was approved by the local Ethics Committee (#12248/2010).

The medical records of 102 patients diagnosed with BP within a period of 35-years were reviewed. They all had been living in Southeastern Brazil. BP diagnosis was based on clinical findings, confirmed by subepidermal blistering upon histopathological examination and by IgG linear fluorescence at the Basement Membrane Zone (BMZ) upon direct and indirect immunofluorescence (DIF and IIF, respectively). Salt-Split-Skin on IIF (SSS-IIF) was performed with anti-IgA, anti-IgG and C`. IgG anti-BP180 and anti-BP230 autoantibodies were determined by ELISA assays (cut-off value = 9 U/mL; MBL, Japan).

For the eight BP-milia patients included herein, the clinical data were assessed, and the following assays were accomplished on stocked DNA and serum samples. For total IgE determination, 50 µL of serum was prepared according to the manufacturer's recommendations (ImmunoCAPTM Total IgE, Thermo-Fisher, USA). Titration was carried out on a Phadia100 laboratory system (Thermo-Fisher, USA). The Immunoblot (IB) assays with BMZ antigens BP180-NC16a and BP180 C-terminal domains, BP230, LAD-1, type VII collagen, and laminin-332 molecules were performed. HLA-DQ alleles were determined by PCR combined with Sequence-Specific Oligonucleotide Probes (PCR-SSOP). The LAB Type kit (One Lambda Inc., Kittredge, CA, USA) was used according to the manufacturer's instructions and Luminex technology.

## Results

**Figure 1:**
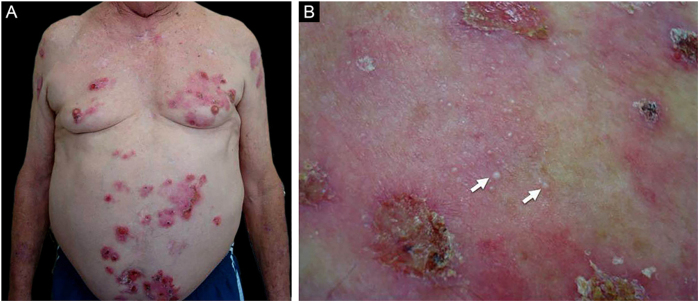
Patient numbered 2 in the Table 1, Table 2. (A), Crusted lesions above erythematous plaques in the anterior thorax and abdomen. (B), Milium lesions (white arrows) over cicatricial blistering lesions can be seen in a high magnification image.

**Figure 2:**
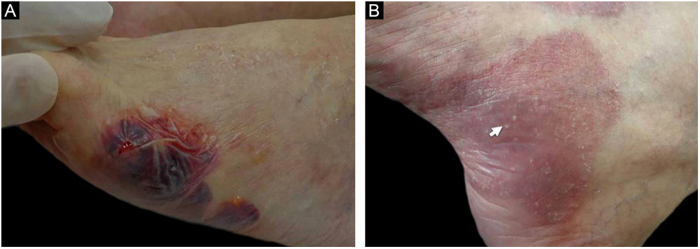
Patient numbered 4 in the Table 1, Table 2. (A), Hemorrhagic bullous and tense vesicle on erythematous plaques in the foot. (B), Milium lesions (white arrow) over cicatricial blistering lesions can be seen.

Of the 102 compiled BP cases, eight exhibited milium lesions (Figure 1, Figure 2), corresponding to BP-milia prevalence of 7.8%. Table 1 summarizes the demographic and clinical data of these eight patients. Five were male, the median age was 75.5-years, and the median disease duration was five months. Clinical description showed active BP lesions in all eight patients. Just one patient presented mucosal involvement. None of them had changed their usual medications before the BP onset. Of interest, four patients presented Neurological Diseases (ND). Seven patients responded well to the usual treatment, but one died due to pulmonary thromboembolism following spinal disc herniation surgery.

**Table 1:** Demographical and clinical data of patients with BP-milia association.

Patient number	Gender	Age (years)	Duration of disease (months)	Distribution of lesions	Medical background and comorbidities	Medications in use at onset of BP lesions
1	Male	83	5	Trunk, limbs, hands, and feet	Recurrent erysipelas, hypertension, dyslipidemia, and resected bladder cancer	Enalapril, amlodipine, simvastatin, carvedilol, and omeprazole
2	Male	71	10	Trunk, abdomen, and limbs	Thyroid disease, hypertension, Diabetes Mellitus (DM) type 2, and history of pulmonary histoplasmosis	Metformin, losartan, aspirin, ramipril, omeprazole, and atorvastatin
3	Male	85	6	Trunk and lower limbs	Hypertension, DM Type 2, and stroke	Aspirin, carvedilol, furosemide, isosorbide dinitrate, omeprazole, NPH, and insulin
4	Female	67	3	Neck, trunk, and limbs	Hypertension, hypothyroidism, hyperparathyroidism, and asthma	Losartan, amlodipine, levothyroxine, ranitidine, duloxetine, pregabalin, primidone, calcium carbonate, magnesium pidolate, potassium chloride, folic acid, and vitamin E
5	Male	80	4	Trunk and hands	Hypertension, DM Type 2, dyslipidemia, and stroke	Hydrochlorothiazide, atenolol, aspirin, phenytoin, metformin, simvastatin, diosmin, indapamide, and cilostazol
6	Male	70	8	Scalp, face, trunk, abdomen, and upper limbs	Hypertension, dyslipidemia, smoker, alcoholism, recurrent urinary infection, stroke, and episode of psychosis	Hydrochlorothiazide, simvastatin, clopidogrel, nortriptyline, and amiodarone
7	Female	46	3	Axillary region, thighs, and feet	Chronic headache	Desogestrel, ethinyl estradiol, and metamizole
8	Female	88	5	Oral mucosae, chest, hands and feet, and inguinal region	Parkinson's disease and dementia	Prolopa, domperidone, omeprazole, doxiciclin, and nicotinamide

**Figure 3:**
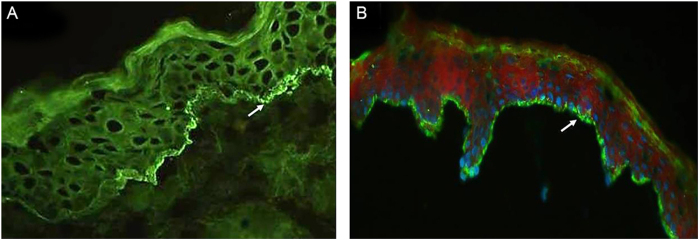
(A), Direct immunofluorescence of skin biopsy showing IgG linear fluorescence in the basement membrane zone (white arrow) (Patient 2) (immunofluorescence, 400×). (B), Indirect immunofluorescence using 1M-NaCl-Salt-split-skin shows IgG fluorescence on the cleavage epidermal side (Patient 5) (immunofluorescence, 400×).

Table 2 details the laboratory findings. Six of seven patients had a subepidermal blister, and eosinophilic inflammatory infiltrate in the upper dermis predominated in four upon histopathological examination. In five of six patients, DIF confirmed IgG or C3, or both, but no IgA linear fluorescence at the BMZ (Fig. 3A). In eight patients, SSS-IIF showed IgG or C3, or both, but no IgA linear fluorescence on the cleavage epidermal side (Fig. 3B). Of interest, increased total serum IgE levels ranging from 252 to >5000 kU/mL were documented in seven of eight patients. Of eight patients, ELISA showed increased anti-BP180, anti-BP230, and both anti-BP180 and anti-BP230 antibody levels in two, one, and three patients, respectively; two patients tested negative for anti-BP180 and anti-BP230 antibodies.

**Table 2:** Laboratory data of patients with BP-milia association.

Patient number	Histopathological features on skin biopsy	DIF	SSS IIF	Serum IgE (kU/mL)^a^	Anti-BP180 (U/mL)^b^	Anti-BP230 (U/mL)^b^	Immunoblot findings	*HLA-DQ* alleles
1	Subepidermal blister with eosinophils within the blister and in the upper dermis.	IgG and C3	IgG and C3	>5000	128.9	137.9	BP180-NC16a domain	NA.
2	Epidermal detachment; inflammatory infiltrate with lymphocytes, neutrophils, and eosinophils in the upper dermis.	C3 and fibrinogen; IgG negative	IgG and C3	>5000	2.3	4.6	BP180 C-terminal-domain and LAD-1	NA.
3	Epidermis exhibiting mild spongiosis. Discreet interstitial lymphocytic inflammatory infiltrate in the upper dermis.	Negative	IgG	782	5.3	1.7	None	NA.
4	Focus of epidermal detachment. Mild perivascular and interstitial inflammatory infiltrate with lymphocytes in the upper dermis.	IgG and C3	IgG and C3	>5000	159.2	72.0	BP180-NC16a domain	DQA1* 01:02/02:01
								DQB1* 02:02/06:02
5	N.A.	IgG and C3	IgG	252	107.3	4.5	BP180-NC16a, BP180 C-terminal-domain, and LAD-1	DQA1* 01:01/05:05
								DQB1* 03:01/05:01
6	Subepidermal blister with fibrin, neutrophils, and lymphocytes. Moderate perianexial, perivascular and interstitial lymphocytic infiltrate in the upper dermis.	Negative	IgG	1286	4.4	68.1	BP230; BP180 C-terminal domain	DQA1* 01:02/02:01
								DQB1* 02:02/06:02
7	Subepidermal blister; inflammatory infiltrate with numerous eosinophils and lymphocytes in the upper dermis.	NA.	IgG	7.1	98.4	1.0	BP180-NC16a, BP180 C-terminal domain, and LAD-1	DQA1* 05:05/05:05
								DQB1* 03:01/03:01
8	Subepidermal vesicle with some eosinophils inside; necrotic keratinocytes in the floor of cleavage. Interstitial and perivascular inflammatory infiltrate with predominance of lymphocytes and eosinophils in the upper dermis.	Negative	IgG and C3	427	169.2	23.3	BP180-NC16a domain and LAD-1	DQA1* 01:03/05:05
								DQB1* 03:01/06:03

BP, Bullous Pemphigoid; DIF, Direct Immunofluorescence with linear Fluorescence deposition along the basement membrane zone; SSS-IIF, Salt Split Skin method on Indirect Immunofluorescence with linear Fluorescence at epidermal cleavage; IgA fluorescence negative. NA, Not available.

a Serum IgE normal range <100 kU/mL.

b Anti-BP180 and anti-BP230 autoantibodies: cut-off 9 U/mL (ELISA, MBL, Japan).

**Figure 4:**
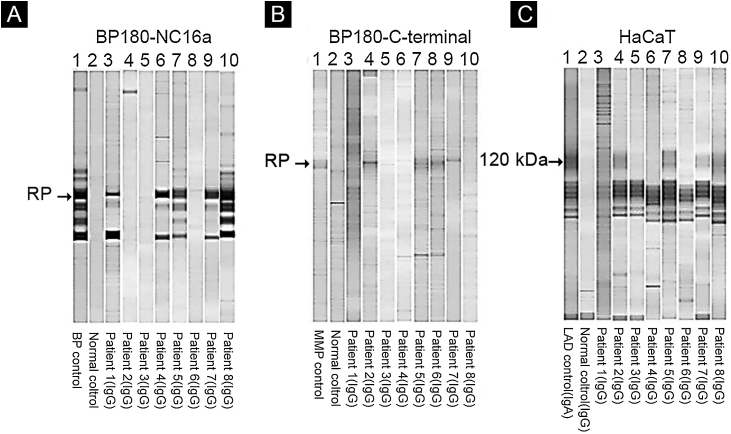
Immunoblot using BP180-NC16a (A), BP180 C-terminal (B), and HaCaT cells (C) with serum samples. For positive results see the RP (reference protein) from: BP (Bullous Pemphigoid) patient's serum (A), MMP, Mucous Membrane Pemphigoid patient's serum (B), and LAD, Linear IgA Dermatosis patient's serum (C).

Of eight patients, IB assays highlighted IgG reactivity against BP230 in one patient (data not shown), against the BP180-NC16a domain in five patients (Fig. 4A), and against the BP180 C-terminal domain or LAD-1, or both, in four patients (Fig. 4B and C). No patient presented reactivity against type VII collagen or laminin-332. We were able to define the HLA-DQ alleles in five of eight patients (see Table 2, last column).

## Discussion

To our best knowledge, BP-milia association is still considered an uncommon clinical finding.^5^, ^9^, ^10^, ^11^, ^12^, ^13^, ^14^, ^15^ There is no report of BP-milia association in a Brazilian population. Here, a prevalence of 7.8% of BP-milia association amongst 102 BP patients is presented, indicating that careful differential diagnosis with EBA is mandatory given that milia formation is frequently reported in EBA.^6^

Due to the increased interest in BP-milia association, we would like to highlight some clinical and laboratory features presented by the eight BP-milia patients. (i) ND association was described in four patients-three with stroke (patients numbered 3, 5 and 6) and one with Parkinson's disease, and dementia (patient 8). ND in association with BP opens an interesting link for BP pathogenesis,^4^, ^16^ but no ND-BP-milia association has been found in the literature. (ii) Increased total IgE levels were demonstrated in seven patients. Increased total IgE has been related to BP severity,^17^, ^18^, ^19^, ^20^ and has been reported in a case of BP-milia association.^13^ The fact that increased total IgE has been identified in 87.5% of the BP-milia patients included herein deserves future confirmation. (iii) Interestingly, four patients showed antibodies against the BP180 C-terminal domain and LAD-1 in IB. The interaction of hemidesmosome proteins and extracellular matrix components beneath hemidesmosomes may also result in milium formation,^5^ but these features are not exclusive of BP-milia association.

Finally, HLA genes are probably the most significant genetic predisposition factor in BP pathogenesis.^2^ In the largest case-series of BP-milia associations to date, milia were present in 23 (31.1%) of 74 British BP patients, and the *HLA*-*DQ*6 allelic group was associated with the BP-milia condition.^21^ Here, three of five cases presented the HLA-DQB1*06 allelic group. Moreover, three exhibited the *HLA*–DQA1*01:03/05:05 alleles, the *HLA*–DQB1*03:01 allele, or all of them, which all represent BP susceptibility alleles in the Brazilian population.^22^

## Conclusion

In conclusion, milium lesions associated with BP seem to be less uncommon than previously admitted, configuring a special differential diagnosis with other blistering diseases. Milium formation in BP deserves a pathogenesis-based explanation as to why certain BP patients develop them.

## Financial support

This study was partially supported by FAPESP (Fundação de Amparo à Pesquisa do Estado de São Paulo), process number 2010/51729-2, and by FAEPA (Fundação de Apoio ao Ensino, Pesquisa e Assistência). SV and TAJ received a PhD scholarship from CAPES (Coordenação de Aperfeiçoamento de Pessoal de Nível Superior), and from FAPESP, respectively.

## Authors' contributions

Sebastián Vernal: Has contributed with collection and interpretation of data, writing the manuscript, effective participation, literature review, final approval of the final version of the manuscript.

Ederson Valei de Oliveira: Has contributed with the collection and interpretation of data, effective participation, final approval of the final version of the manuscript.

Roberto Bueno Filho: Has contributed with the collection and interpretation of data, effective participation, participation of therapeutic conduct of the studied cases, final approval of the final version of the manuscript.

Tamiris A. Julio: Has contributed with the collection and interpretation of data, effective participation, final approval of the final version of the manuscript.

Eduardo A. Donadi: Has contributed with the collection and interpretation of data, effective participation, final approval of the final version of the manuscript.

Aline Turatti: Has contributed with the collection and interpretation of data, effective participation, final approval of the final version of the manuscript.

Norito Ishii: Has contributed with the collection and interpretation of data, effective participation, final approval of the final version of the manuscript.

Takashi Hashimoto: Has contributed with the collection and interpretation of data, effective participation, writing the manuscript, final approval of the final version of the manuscript.

Ana Maria Roselino: Has contributed with the study concept and design, collection, and interpretation of data, writing the manuscript, effective participation, literature review, participation of therapeutic conduct of the studied cases, final approval of the final version of the manuscript.

## Conflicts of interest

None declared.
